# *Arthroplasty*: a new platform for sharing advances in arthroplasty and joint diseases

**DOI:** 10.1186/s42836-019-0005-5

**Published:** 2019-07-31

**Authors:** Yan Wang

**Affiliations:** Department of Orthopedics, Chinese PLA General Hospital, Beijing, China

Over the past decades, many new techniques, tools, and methodologies have been developed rapidly in all aspects of joint surgery, ranging from clinical and basic research to translational medicine of joint diseases, and arthroplasty. In those fields, the cooperation among the countries, continents, and regions has become even closer. Surgeons, therapists, and researchers around the world have more desire to share innovative ideas and exciting experience with others. Therefore, a platform is needed to connect the thoughts and practices, to generate more opportunities to articulate ideas and views on joint surgery and the related sciences.

The journal, *Arthroplasty*, is a collaborative result of the Arthroplasty Society in Asia (ASIA) and BMC. It aims to exhibit insightful discoveries by publishing high-quality original articles and reviews. The goal is to provide a platform for researchers and academics all over the world to promote, share and discuss various new issues and developments in joint replacement, joint diseases, prosthetic design, and related problems. For more details of the aims and scope of the journal, please check out the website: https://arthroplasty.biomedcentral.com.

The Editorial Board of *Arthroplasty* consists of a group of international consultants and specialists to provide the authors with unbiased, constructive, and timely peer reviews and to help disseminate their research findings expeditiously and broadly. All contributions will be published online promptly after acceptance and proofreading. We truly appreciate the efforts of all authors whose manuscripts could make space in this brand-new journal. We thanks all members of the editorial board, review board, contributors, colleagues, and staff members who have extended their supports that made the first issue of *Arthroplasty* possible.

We are publishing several reviews and full research articles to mark the launch of Arthroplasty.

In the research article entitled “Treatment of periprosthetic hip infection with retention of a well-fixed stem: six to 13-year outcomes”, Takuya Otani and colleagues reported that the two-stage treatment was a beneficial strategy to minimize the local and systemic burden of the patient. They successfully enhanced the functional preservation of the hip joint while achieved infection control in the long term.

In the review entitled “Reducing the risk of infection after total joint arthroplasty: preoperative optimization”, Antonelli and Chen reviewed the recent progresses on the specific modifiable risk factors associated with surgical site infection and periprosthetic hip infection. They advocated that patients who had one or more of the risk factors (MSSA/MRSA colonization, rheumatoid arthritis, cardiovascular and renal disease, obesity, diabetes mellitus, hyperglycemia, anemia, malnutrition, tobacco use, alcohol consumption, depression, and anxiety) should be given a multidisciplinary intervention including patient education, counseling, and follow-up. The preoperative intervention could optimize patients to decrease the risk of total joint arthroplasty.

Total hip arthroplasty for the developmental dysplasia of the hip is still a technically demanding surgery that requires an in-depth understanding of the anatomical abnormalities and complex techniques. Professor Yan Wang, the editor-in-chief, presents a review entitled “Current concepts in developmental dysplasia of the hip and total hip arthroplasty”. In this review, Professor Wang introduces the key issues of acetabular and femoral reconstruction in total hip arthroplasty for dysplasia of the hip. Those issues included the high of hip center, acetabular bone deficiency, highly porous metal, correction of femoral anteversion, femoral shortening osteotomy, and selection of stems. We hope those issues will stir up a further discussion in this important field to advance acetabular and femoral reconstruction.

We hope you enjoy reading the first series of papers, and cordially invite you to share your experience, discoveries, and insights with the readers. We look forward to receiving your submissions soon.

